# Diagnostic accuracy of symptoms as a diagnostic tool for SARS-CoV 2 infection: a cross-sectional study in a cohort of 2,173 patients

**DOI:** 10.1186/s12879-021-05930-1

**Published:** 2021-03-11

**Authors:** Carlos Alfonso Romero-Gameros, Tania Colin-Martínez, Salomón Waizel-Haiat, Guadalupe Vargas-Ortega, Eduardo Ferat-Osorio, José Alberto Guerrero-Paz, Marielle Intriago-Alor, Mayra Alejandra López-Moreno, Carlos Fredy Cuevas-García, Victoria Mendoza-Zubieta, Jose Luis Martínez-Ordaz, Baldomero González-Virla

**Affiliations:** 1 Otorhinolaryngology Service, Hospital de Especialidades, Centro Médico Nacional Siglo XXI, Instituto Mexicano del Seguro Social, Mexico City, Mexico; 2 Emergency Department. Hospital de Especialidades, Centro Médico Nacional Siglo XXI, Instituto Mexicano del Seguro Social, Mexico City, Mexico; 3 Endocrinology Service, Hospital de Especialidades, Centro Médico Nacional Siglo XXI, Instituto Mexicano del Seguro Social, 330 Cuauhtémoc Avenue, 06720 Mexico City, Mexico; 4 Education and Research Division. Hospital de Especialidades, Centro Médico Nacional Siglo XXI, Instituto Mexicano del Seguro Social, Mexico City, Mexico; 5 General Director of the Hospital de Especialidades, Centro Médico Nacional Siglo XXI, Instituto Mexicano del Seguro Social, Mexico City, Mexico

**Keywords:** COVID-19, Diagnostic accuracy, SARS-CoV-2, Positive predictive value, Symptomatology

## Abstract

**Background:**

The SARS-CoV-2 pandemic continues to be a priority health problem; According to the World Health Organization data from October 13, 2020, 37,704,153 confirmed COVID-19 cases have been reported, including 1,079,029 deaths, since the outbreak. The identification of potential symptoms has been reported to be a useful tool for clinical decision-making in emergency departments to avoid overload and improve the quality of care. The aim of this study was to evaluate the performances of symptoms as a diagnostic tool for SARS -CoV-2 infection.

**Methods:**

An observational, cross-sectional, prospective and analytical study was carried out, during the period of time from April 14 to July 21, 2020. Data (demographic variables, medical history, respiratory and non-respiratory symptoms) were collected by emergency physicians. The diagnosis of COVID-19 was made using SARS-CoV-2 RT-PCR. The diagnostic accuracy of these characteristics for COVID-19 was evaluated by calculating the positive and negative likelihood ratios. A Mantel-Haenszel and multivariate logistic regression analysis was performed to assess the association of symptoms with COVID-19.

**Results:**

A prevalence of 53.72% of SARS-CoV-2 infection was observed. The symptom with the highest sensitivity was cough 71%, and a specificity of 52.68%. The symptomatological scale, constructed from 6 symptoms, obtained a sensitivity of 83.45% and a specificity of 32.86%, taking ≥2 symptoms as a cut-off point. The symptoms with the greatest association with SARS-CoV-2 were: anosmia odds ratio (OR) 3.2 (95% CI; 2.52–4.17), fever OR 2.98 (95% CI; 2.47–3.58), dyspnea OR 2.9 (95% CI; 2.39–3.51]) and cough OR 2.73 (95% CI: 2.27–3.28).

**Conclusion:**

The combination of ≥2 symptoms / signs (fever, cough, anosmia, dyspnea and oxygen saturation < 93%, and headache) results in a highly sensitivity model for a quick and accurate diagnosis of COVID-19, and should be used in the absence of ancillary diagnostic studies. Symptomatology, alone and in combination, may be an appropriate strategy to use in the emergency department to guide the behaviors to respond to the disease.

**Trial registration:**

Institutional registration R-2020-3601-145, Federal Commission for the Protection against Sanitary Risks 17 CI-09-015-034, National Bioethics Commission: 09 CEI-023-2017082.

## Background

The SARS-CoV-2 pandemic continues to be a priority health problem. According to figures of the WHO, as of October 13, 2020; 37,704,153 confirmed cases have been reported, including 1,079,029 deaths, since the outbreak. The majority were concentrated in the region of the Americas, representing 48% of cases and 55% of total deaths [1]. In Mexico, since the first case was confirmed, to November 1, 2020; a total of 929,392 cases and 91,895 deaths have been reported [2].

It have been determined that the peak of infectiousness occurs in the early stages, so timely interventions plays an important role in controlling its spread [3, 4]. Currently, diagnostic tools for SARS-CoV-2 infection, include methods based on viral nucleic acid detection, antigen-antibody reaction tests and imaging studies such as chest computed tomography [5, 6]. However, in low income countries, where human and material resources are limited, the use of algorithms based on clinical data has been suggested to help correctly identify patients with a high probability for COVID-19 and thus achieve the resource optimization [7]. Symptom evaluation have been reported to be a useful tool for clinical decision-making in emergency departments, to avoid work overload and to improve the quality of care [8, 9].

In July of this year and with the aim of assessing the diagnostic accuracy of signs and symptoms, *Struyf* et al. conducted a systematic review of 16 studies (including 7706 patients). The authors concluded that the evaluation of signs and symptoms (individually) has little value for the diagnosis of SARS-CoV-2, and thus emphasized the urgent need of prospective clinical studies which evaluate the combination of signs and symptoms, in search of greater specificity for the detection of the SARS-CoV-2 infection [10].

The aim of the study was to evaluate and establish the diagnostic performance of the symptoms and signs (isolated and in combination) in patients with suspected COVID-19, as a screening tool for the correct identification of positive cases.

## Material and methods

An observational, transversal, prospective and analytical study was carried out at the *Hospital de Especialidades del Centro Médico Nacional Siglo XXI* of the Social Security Mexican Institute IMSS (a tertiary care center), in Mexico City, Mexico; during the period from April 14 to July 21, 2020, with patients who arrived at the emergency area due to COVID-19 suspicion.

This study was approved by the National Commission on Bioethics (09 CEI-023-2,017,082), the Federal Commission for Protection against Health Risks Research (17 CI-09-015-034) and the local hospital research committee (R-2020-3601-145).

### Data collection

The study included patients who came to the emergency department for medical attention due to suspected COVID-19. Patients were selected through a non-probabilistic sampling of consecutive cases according to the order of arrival at the emergency department. The patients were evaluated and selected by 6 emergency physicians who, according to the guidelines established by the Mexican General Directorate of Epidemiology [11], indicated the collection of a sample for detection of SARS-CoV-2 by RT-PCR.

The data were collected through the application of a questionnaire consisting of 30 variables, applied by six emergency physicians during the assessment in the emergency area, in which demographic variables (age, gender) were included; as well as their medical history (comorbidities, vital signs, anthropometry), respiratory and non-respiratory symptoms related to the SARS-CoV-2 infection (fever, cough, odynophagia, chest pain, asthenia, myalgia, rhinorrhea, headache, anosmia, conjunctivitis, and dyspnea). Fever was defined as the presence of a body temperature greater than 38 °C (100.4 °F).

The patients had to meet the following inclusion criteria in order to be able to participate: patients over 17 years of age of both genders, who signed an informed consent form, patients with a high clinical probability of SARS-CoV-2 infection; and who had confirmatory SARS-CoV-2 RT-PCR.

**Fig. 1:**
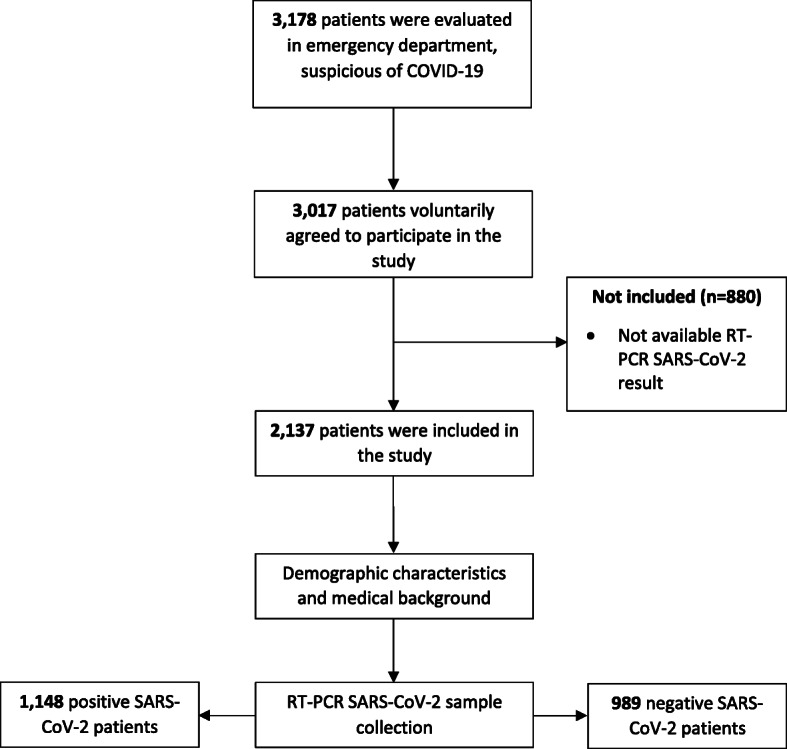
Study flowchart

Patients who did not have a SARS-CoV-2 RT-PCR result and who did not sign an informed consent form were excluded Fig. 1.

### Diagnosis of SARS-CoV-2

The decision to test for SARS-CoV-2 was made according to the clinical judgment of the emergency physician in charge. The identification of SARS-CoV-2 was performed by real time RT-PCR, in samples of nasopharyngeal exudate which were sent to the Central Laboratory of Epidemiology of the National Medical Center “*La Raza*” for their processing, following the international standards for the transport of infectious substances. The sample processing for the detection of SARS-CoV-2 was performed according to the guidelines certified by the National Institute for Diagnosis and Epidemiological Referral [12].

### Construction of the symptom diagnostic scale

A combination of the symptomatology related to the SARS-CoV-2 infection was assessed to calculate the association and magnitude of it with the presence of COVID-19, to construct the diagnostic scale. Symptom selection was made according to a model obtained through a LASSO regression.

### Statistical analysis

Descriptive and inferential statistics were used for the data analysis, taking into account measures of central tendency and dispersion. For the comparison of frequencies and proportions, a chi-square statistical test was used. To compare the quantitative variables, we used the Mann-Whitney’s U statistical test, or T test according to the distribution of the variables. The Kolmogorov-Smirnov test was used to determine the normality in the variable’s distribution.

A stratified Mantel-Haenszel analysis was performed to assess the association of symptoms to the probability of a SARS-CoV-2 positive result. For each symptom, sensitivity and specificity, positive likelihood ratio (LR+), negative likelihood ratio (LR-), accuracy, and area under the curve (AUC) were calculated, as a diagnostic tool for identifying the SARS-CoV 2 infection. A multiple logistic regression analysis was performed, including symptoms -alone and in combination- related to SARS-CoV-2 infection, adjusted for age, gender, and comorbidities, to estimate the magnitude of the association. A LASSO regression analysis was used to establish the symptomatology of a SARS-CoV2 infection. A value of *P* < 0.05 was considered statistically significant. The statistical program used was SPSS, version 25.0 (IBM SPSS Statistics for Windows. Armonk, NY: IBM Corp) and the Stata SE software, version 16 (StataCorp, TX, USA).

## Results

### Baseline characteristics of patients with SARS-CoV-2 infection

During the study period, a total of 3178 patients were evaluated in the emergency area for suspected COVID-19. 3017 patients voluntarily agreed to participate in the study; 880 patients did not have a SARS-CoV-2 RT-PCR test, because they didn’t meet the definition of a suspected case, according to the guidelines of the Mexican General Directorate of Epidemiology [11], so they were not included. At the end, a total of 2137 patients were included; 1148 (53.72%) were positive for SARS-CoV-2; of the SARS-CoV-2 positive group, 508 (44.25%, *P* < 0.001) were female, with an average age of 48.60 (SD ± 15.6, *P* < 0.001) Table 1.

**Table 1:** Baseline characteristics and clinical history of 2137 patients presenting for COVID-19 suspicion

Variable	Total (***n*** = 2137)	Positive SARS CoV2 patients (***n*** = 1148)	Negative SARS CoV2 patients (***n*** = 989)	***P*** Value
Age, mean ± SD	45.93 ± 15.34	48.60 ± 15.6	42.82 ± 14.42	< 0.001^a^
**Gender, n (%)**				
Female	1034 (48.3)	508 (44.2)	526 (53.1)	< 0.001^b^
Male	1103 (51.6)	640 (55.7)	463 (46.8)	
**Vital sings**				
Temperature (C°), median (IQR)	36.4 (36.2)	36.4 (36.2–36.8)	36.4 (36.2–36.7)	0.01^c^
Hear rate (bpm), median (IQR)	90 (78–103)	94 (82–108)	87 (75–99)	< 0.001^c^
Respiratory rate (bpm), median (IQR)	22 (20–24)	22 (20–24)	22 (20–24)	< 0.001^c^
SBP (mmHg), mean ± SD	191.2 ± 19.1	131.3 ± 18.6	131.1 ± 19.7	0.78^a^
DBP (mmHg), mean ± SD	76.6 ± 20.0	76.7 ± 24.4	76.5 ± 13.2	0.82^a^
Oxygen saturation (%), median (IQR)	94 (90–95)	93 (85–95)	95 (93–96)	< 0.001^c^
Weight (kg), median (IQR)	74 (63–85)	75 (64–86)	72 (62–84)	< 0.001^c^
Height (m), mean ± SD	1.63 ± 0.09	1.62 ± 0.09	1.63 ± 0.09	0.42^a^
BMI (kg/m^2^), median (IQR)	27.8 (24.76–31.21)	28.4 (25.28–31.74)	27.3 (24.11–30.46)	< 0.001^c^
Oxygen saturation < 93%, n (%)	170 (7.96)	127 (11.06)	43 (4.35)	< 0.001 ^a^
**General symptoms, n (%)**				
Fever	891 (41.69)	615 (53.57)	276 (27.91)	< 0.001^b^
Cough	1284 (60.08)	816 (71.08)	468 (47.32)	< 0.001 ^b^
Odynophagia	1058 (49.51)	574 (50.00)	484 (48.94)	0.62 ^b^
Thoracic pain	708 (33.13)	445 (38.76)	263 (26.59)	< 0.001 ^b^
Asthenia	1354 (63.36)	789 (68.73)	565 (57.13)	< 0.001 ^b^
Myalgia	1188 (55.59)	705 (61.41)	483 (48.84)	< 0.001 ^b^
Rhinorrhea	652 (30.51)	373 (32.49)	279 (28.21)	0.03 ^b^
Headache	1287 (60.22)	709 (61.76)	578 (58.44)	0.11 ^b^
Anosmia	410 (19.19)	309 (26.92)	101 (10.21)	< 0.001 ^b^
Conjunctivitis	304 (14.23)	162 (14.11)	142 (14.36)	0.87 ^b^
Dyspnea	788 (36.87)	550 (47.91)	238 (24.06)	< 0.001 ^b^
**Comorbidities, n (%)**				
COPD	29 (1.36)	21 (1.83)	8 (0.81)	0.04 ^b^
Type 2 diabetes mellitus	326 (15.26)	218 (18.99)	108 (10.92)	< 0.001 ^b^
Hypertension	447 (20.92)	285 (24.83)	162 (16.38)	< 0.001 ^b^
Chronic Kidney disease	84 (3.93)	54 (4.78)	30 (3.03)	0.04 ^b^
Immunodeficiency	65 (3.04)	34 (2.96)	31 (3.13)	0.81 ^b^
Hepatopathy	11 (0.51)	8 (0.70)	3 (0.30)	0.20 ^b^
Obesity	1148 (57.72)	537 (60.47)	611 (48.92)	< 0.001 ^b^

*Abbreviations: IQR* interquartile range, *SD* standard deviation, *Kg* kilograms, *BMI* body mass index, *m* meters, *COPD* Chronic Obstructive Pulmonary Disease

^a^ *P* Value estimated with T test between SARS-CoV2 positive and SARS-CoV2 negative patients

^b^ *P* Value estimated with Pearson’s X^2^ test between SARS-CoV2 positive and SARS-CoV2 negative patients

^c^ *P* Value estimated with U-Mann-Whitney between SARS-CoV2 positive and SARS-CoV2 negative patients

The following vital signs were obtained at the time of evaluation in the emergency room: heart rate of 94 beats per minute (IQR; 82–108, *P* < 0.001), respiratory rate of 22 breaths per minute (IQR; 20–24, *P* < 0.001), and pulse oximetry oxygen saturation of less than 93% (SO2 < 93%) (IQR; 85–95, *P* < 0.001), mean systolic blood pressure of 131 mmHg (SD ± 18.67, *P* = 0.78), and diastolic blood pressure of 76 mmHg (SD ± 24.42, *P* = 0.82) Table 1.

### Symptoms associated with SARS-CoV-2 infection

In the patients assessed, the most frequent symptom was cough in 71.08% (*P* < 0.001), followed by asthenia in 68.73% (*P* < 0.001), headache in 67.76% (*P* = 0.11), myalgia in 61.4% (*P* < 0.001), fever in 53.57% (*P* < 0. 001), odynophagia in 50% (*P* = 0.62), dyspnea in 47.91% (*P* < 0.001), chest pain in 38.76% (*P* < 0.001), rhinorrhea in 32.49 (*P* = 0.03) and anosmia in 26.92% (*P* < 0.001); the least frequent being conjunctivitis, in 14.11% (*P* = 0.87) Table 1.

### Comorbidities in the SARS-CoV-2 positive patient group

The most frequent comorbidity was obesity (60.47%, *P* < 0.001), followed by hypertension (24.38%, *P* < 0.001), type 2 diabetes mellitus (18.99%, *P* < 0.001) and chronic renal disease (4.78%, *P* = 0.04) Table 1.

### Stratified and multivariate analysis of the symptomatology of SARS-CoV-2 positive patients

**Fig. 2:**
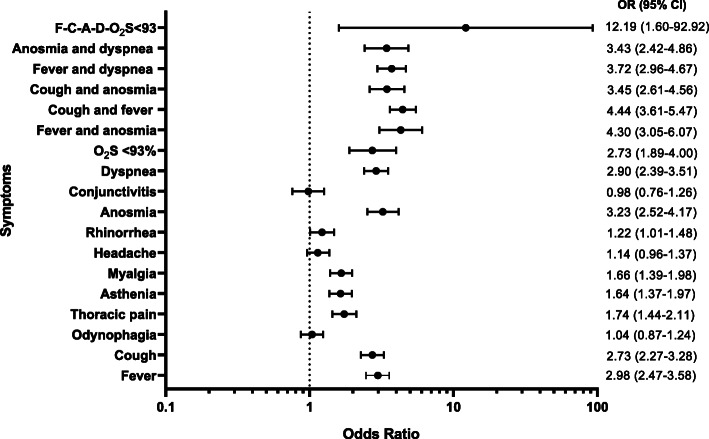
Stratified analysis of symptomatology of 2137 patients presenting for COVID-19 suspicion

Anosmia presented an odds ratio (OR) of 3.2 ([95% CI: 2.52–4.17], *P* < 0.001), followed by fever with an OR of 2.98 ([95% CI: 2.47–3.58], *P* < 0.001), dyspnea with OR of 2. 9 ([95% CI: 2.39–3.51], *P* < 0.001), cough with OR of 2.73 ([95% CI: 2.27–3.28], *P* < 0.001) and oxygen saturation < 93% OR of 2.73 ([95% CI: 1.89–4], *P* < 0.001) Table 2 and Fig. 2.

**Table 2:** Stratified and multivariate analysis of symptomatology of 2137 patients presenting for COVID-19 suspicion

	Stratified analysis			Multivariate analysis^**b**^		
Variable	OR	95% CI	***P*** vale^a^	OR	95% CI	***P*** vale^a^
**Fever**	2.98	2.47–3.58	< 0.001	1.96	1.58–2.41	< 0.001
**Cough**	2.73	2.27–3.28	< 0.001	1.95	1.58–2.41	< 0.001
Odynophagia	1.04	0.87–1.24	0.62			
Thoracic pain	1.74	1.44–2.11	< 0.001			
Asthenia	1.64	1.37–1.97	< 0.001			
**Myalgia**	1.66	1.39–1.98	< 0.001	1.25	1.00–1.56	0.04
Headache	1.14	0.96–1.37	0.11			
Rhinorrhea	1.22	1.01–1.48	0.03			
**Anosmia**	3.23	2.52–4.17	< 0.001	2.96	2.27–3.87	< 0.001
Conjunctivitis	0.98	0.76–1.26	0.87			
**Dyspnea**	2.90	2.39–3.51	< 0.001	1.48	1.17–1.87	0.001
**O**_**2**_**S < 93**	2.73	1.89–4.00	< 0.001	1.56	1.17–1.87	0.001
Fever and anosmia	4.30	3.05–6.07	< 0.001			
**Cough and fever**	4.44	3.61–5.47	< 0.001	2.79	2.12–3.69	< 0.001
**Cough and anosmia**	3.45	2.61–4.56	< 0.001	2.48	1.65–3.74	< 0.001
Fever and dyspnea	3.72	2.96–4.67	< 0.001			
Anosmia and dyspnea	3.43	2.42–4.86	< 0.001			
Fever, cough, anosmia, dyspnea, O_2_S < 93	12.19	1.60–92.92	< 0.001			

*Abbreviations: OR* odds ratio, *O*_*2*_*S* Oxygen saturation, *95% CI* 95% confidence interval

^a^ *P* Value estimated with Pearson’s X2 test

^b^ Multivariate logistic regression of symptomatology adjusted by age, gender and comorbidities

An OR of 12.13 ([95% CI: 1.6–92.92], P < 0.001) was obtained for the combination of fever, cough, anosmia, dyspnea, and oxygen saturation of less than 93% (SO2 < 93%); similarly, for the cough-fever combination, with an OR of 4. 44 ([95% CI: 3.61–5.47], *P* < 0.001), and for fever-anosmia, with an OR of 4.3 ([95% CI: 3.05–6.07], *P* < 0.001). Table 2 and Image 2.

In a multivariate logistic regression model where isolated and combined symptoms were included, adjusted for age, gender and comorbidities; anosmia had an OR of 2. 96 ([95% CI: 2.27–3.87], *P* < 0.001), followed by the combination of cough-fever with an OR 2.79 ([95% CI: 2.12–3.69], *P* < 0.001) and cough-anosmia with an OR of 2.48 ([95% CI: 1.65–3.74], *P* < 0.001) Table 2.

### Diagnostic performance of the symptomatology, as a tool for SARS-CoV-2 identification

The three combinations of symptoms with more specificity were: fever-cough-anosmia-dyspnea-SO2 < 93%, fever-anosmia and anosmia-dyspnea. The symptoms with greater sensitivity were cough, asthenia, and headache. See Table 3.

**Table 3:** Diagnostic accuracy of symptoms as an instrument for detection of SARS-CoV-2 infection

Symptoms	Sensitivity	Specificity	PPV %	NPV %	LR+	LR-	Accuracy	***P***^***a***^	AUC
Fever % (95% CI)	53.57 [50.64–56.49]	72.09 [69.18–74.87]	69.02 [66.54–71.40]	57.22 [55.42–59.01]	1.92 [1.71–2.15]	0.64 [0.60–0.69]	62.14 [60.05–64.21]	< 0.001	0.62 0.60–0.64
Cough % (95% CI)	71.08 [68.36–73.69]	52.68 [49.51–55.83]	63.55 [61.79–65.28]	61.08 [58.48–63.62]	1.50 [1.39–1.62]	0.55 [0.49–0.61]	62.56 [60.47–64.62]	< 0.001	0.61 0.59–0.63
Odynophagia % (95% CI)	50.00 [47.07–52.93]	51.06 [47.9–54.22]	54.25 [52.11–56.38]	46.80 [44.72–48.9]	1.02 [0.94–1.11]	0.98 [0.90–1.07]	50.46 [48.35–52.63]	0.62	0.5 0.48–0.52
Thoracic pain % (95% CI)	38.76 [35.93–41.65]	73.41 [70.54–76.14]	62.85 [59.85–65.76]	50.80 [49.32–52.29]	1.46 [1.28–1.65]	0.83 [0.79–0.89]	54.8 [52.66–56.92]	< 0.001	0.56 0.54–0.58
Asthenia % (95% CI)	68.73 [65.96–71.40]	42.87 [39.76–46.02]	58.27 [56.64–59.88]	54.15 [51.36–56.91]	1.20 [1.13–1.29]	0.73 [0.65–0.82]	56.76 [54.63–58.88]	< 0.001	0.55 0.53–0.57
Myalgia % (95% CI)	61.41 [58.53–64.24]	51.16 [48.00–54.32]	59.34 [57.43–61.22]	53.32 [50.95–55.68]	1.26 [1.16–1.36]	0.75 [0.69–0.83]	56.67 [54.54–58.78]	< 0.001	0.56 0.54–0.58
Headache % (95% CI)	61.76 [58.88–64.58]	41.56 [38.46–44.70]	55.09 [53.36–56.08]	48.35 [45.76–50.96]	1.06 [0.99–1.13]	0.92 [0.83–1.02]	52.41 [50.27–54.55]	0.11	0.51 0.49–0.53
Rhinorrhea % (95% CI)	32.49 [29.79–35.29]	71.79 [68.87–74.58]	57.21 [54.01–60.35]	47.81 [46.42–49.21]	1.15 [1.01–1.31]	0.94 [0.89–0.99]	50.68 [48.54–52.82]	0.03	0.52 0.50–0.54
Anosmia % (95% CI)	26.92 [24.37–29.58]	89.79 [87.73–91.60]	75.37 [71.31–79.02]	51.42 [50.40–52.44]	2.64 [2.14–3.24]	0.81 [0.78–0.85]	56.01 [53.88–58.13]	< 0.001	0.58 0.56–0.59
Conjunctivitis % (95% CI)	14.11 [12.15–16.26]	85.64 [83.3–87.77]	53.29 [48.08–58.43]	46.21 [45.35–47.07]	0.98 [0.80–1.21]	1.00 [0.97–1.04]	47.22 [45.08–49.36]	0.87	0.49 0.48–0.51
Dyspnea % (95% CI)	47.91 [44.98–50.84]	75.94 [73.15–78.57]	69.80 [67.07–72.39]	55.67 [54.05–57.28]	1.99 [1.76–2.26]	0.69 [0.64–0.73]	60.88 [58.77–62.96]	< 0.001	0.61 0.59–0.63
O_2_S < 93% (95% CI)	11.06 [9.31–13.02]	95.65 [94.19–96.84]	74.71 [67.87–80.51]	48.09 [47.49–48.70]	2.54 [1.82–3.56]	0.93 [0.91–0.95]	50.21 [48.07–52.35]	< 0.001	0.53 0.52–0.54
**Combination of symptoms**									
Fever and anosmia % (95% CI)	16.38 [14.28–18.65]	95.65 [94.19–96.84]	81.39 [76.04–85.76]	49.63 [48.91–50.35]	3.77 [2.73–5.19]	0.87 [0.85–0.90]	53.07 [50.92–55.20]	< 0.001	0.56 0.54–0.57
Cough and fever, % (95% CI)	44.86 [41.96–47.79]	84.53 [82.12–86.73]	77.10 [74.16–79.78]	56.91 [55.47–58.34]	2.89 [2.47–3.40]	0.65 [0.62–0.69]	63.22 [61.13–65.27]	< 0.001	0.64 0.62–0.66
Cough and anosmia % (95% CI)	21.34 [19.00–23.83]	92.72 [90.92–94.26]	77.29 [72.63–81.35]	50.38 [48.51–51.26]	2.93 [2.29–3.76]	0.85 [0.82–0.88]	54.38 [52.24–56.50]	< 0.001	0.57 0.55–0.58
Fever and dyspnea, % (95% CI)	33.54 [30.81–33.35]	88.07 [85.88–90.02]	76.54 [73.00–79.75]	53.30 [52.13–54.47]	2.81 [2.33–3.39]	0.75 [0.72–0.79]	58.77 [56.65–60.87]	< 0.001	0.60 0.59–0.62
Anosmia and dyspnea, % (95% CI)	13.50 [11.58–15.62]	95.65 [94.19–96.84]	78.28 [72.22–83.33]	48.79 [48.13–49.45]	3.11 [2.24–4.31]	0.90 [0.88–0.93]	51.52 [49.38–53.66]	< 0.001	0.54 0.53–0.55
Fever, cough, anosmia, dyspnea, O_2_S < 93, % (95% CI)	1.22 [0.67–2.04]	99.90 [94.44–100]	93.33 [64.84–99.07]	46.56 [46.39–46.73]	12.06 [1.59–91.56]	0.99 [0.98–1]	46.89 [44.75–49.03]	0.002	0.50 0.50–0.50

*Abbreviations: PPV* positive predictive value, *NPV* negative predictive value, *LR+* positive likelihood ratio, *LR-* negative likelihood ratio, *AUC* area under the curve, *95% CI* 95% confidence interval

^***a***^*P* Value estimated with Pearson’s X^2^ test

### Performance of the symptoms scale as a diagnostic instrument for SARS-CoV-2

Table 4 shows the sensitivity, specificity, LR+, LR-, accuracy test and AUC of the symptomatology scale in which the following symptoms (according to the predictability of the independent variables through LASSO regression) were included: fever, cough, anosmia, dyspnea, SO2 < 93% and headache. The highest sensitivity was obtained with the presence of two or more symptoms, 83.45% ([95% CI: 81.17–85.55%], *P* < 0.001), specificity of 32.86% ([95% CI: 29.94–35.89%], *P* < 0.001).

**Table 4:** Diagnostic accuracy of the diagnostic symptoms scale*

Numbers of symptoms	Sensitivity %	Specificity %	PPV %	NPV %	LR+	LR-	Accuracy	***P***^***a***^	AUC
≥ 2 symptoms [95% CI]	83.45 [81.17–85.55]	32.86 [29.94–35.89]	59.06 [57.83–60.28]	63.11 [59.37–66.69]	1.24 [1.18–1.31]	0.50 [0.43–0.59]	60.04 [57.92–62.2]	< 0.001	0.58 [0.56–0.60]
≥ 3 symptoms [95% CI]	67.39 [64.59–70.10]	58.24 [55.10–61.34]	65.18 [63.25–67.06]	60.63 [58.26–62.96]	1.61 [1.48–1.76]	0.56 [0.51–0.62]	63.16 [61.07–65.21]	< 0.001	0.62 [0.60–0.64]
≥ 4 symptoms [95% CI]	49.69 [46.76–52.63]	80.08 [77.45–82.53]	76.32 [71.60–76.86]	57.85 [56.25–59.44]	2.49 [2.17–2.86]	0.63 [0.59–0.67]	63.76 [61.68–65.81]	< 0.001	0.64 [0.62–0.66]
≥ 5 symptoms % (95% CI)	27.99 [25.4–30.68]	93.23 [91.48–94.71]	82.73 [78.88–86.01]	52.75 [51.75–53.74]	4.13 [3.22–5.30]	0.77 [0.74–0.80]	58.19 [56.07–60.30]	< 0.001	0.60 [0.58–0.62]
≥ 6 symptoms % (95% CI)	11.16 [9.40–13.13]	97.67 [96.53–98.52]	84.77 [78.26–89.59]	48.66 [48.10–49.23]	4.8 [3.10–7.42]	0.91 [0.89–0.93]	51.22 [49.07–53.36]	< 0.001	0.54 [0.52–0.56]

*Abbreviations: PPV* positive predictive value, *NPV* negative predictive value, *LR+* positive likelihood ratio, *LR-* negative likelihood ratio, *OR* odds ratio, *95% CI* 95% confidence interval

* Symptoms scale that included Fever, cough, anosmia, dyspnea and oxygen saturation < 93%, and headache

^a^*P* Value estimated with Pearson’s X^2^ test

### Stratified and multivariate analysis of the symptoms scale for the diagnosis of SARS-CoV-2 with positive patients

The stratified analysis found an OR of the combination of ≥4 symptoms of 3.97 ([95% CI: 3.25–4.84], *P* < 0.001); and of the combination of ≥5 symptoms of 5.34 ([95% CI: 4.02–7.17], *P* < 0.001). With the adjustment of the symptomatology scale by age and gender, the OR obtained was, for ≥4 symptoms and for ≥5 symptoms, of 2.01 ([95% CI: 1.15–2.69], *P* < 0.001) and of 2.16 ([95% CI: 1.45–3.20], *P* < 0.001), respectively Table 5.

**Table 5:** Stratified and multivariate analysis of diagnostic symptoms scale*

	Stratified analysis			Multivariate analysis^**b**^		
Numbers of symptoms	OR	95% CI	***P*** ^**a**^	OR	95% CI	***P*** ^**a**^
≥ 2 symptoms	2.46	2.00–3.04	< 0.001	1.28	0.98–1.67	0.06
≥ 3 symptoms	2.88	2.40–3.45	< 0.001	1.23	0.93–1.62	0.13
≥ 4 symptoms	3.97	3.25–4.84	< 0.001	2.01	1.15–2.69	< 0.001
≥ 5 symptoms	5.34	4.02–7.17	< 0.001	2.16	1.45–3.20	< 0.001
≥ 6 symptoms	5.27	3.32–8.68	< 0.001	1.22	0.70–2.14	0.46

*Abbreviations: OR* odds ratio, *95% CI* 95% confidence interval

* Symptoms scale that included Fever, cough, anosmia, dyspnea and oxygen saturation < 93% and headache

^a^*P* Value estimated with Pearson’s X^2^ test

^b^ Multivariate analysis adjusted by age and gender

## Discussion

The purpose of this study was to evaluate the diagnostic performance of the symptomatology in detecting the SARS-CoV-2 infection. Recently, we reported the diagnostic accuracy of smell disorders,detected by psychophysical tests and by the application of a self-perception questionnaire, in the Mexican population (sensitivity of 19.44% and specificity 95.52% [*P* = 0.007]; sensitivity of 50% and specificity 80.59% [*P* < 0.001], respectively) [13]. However, so far there are no studies done within the Mexican population to evaluate the above-mentioned objective.

In Mexico, some social conditions have been observed that cause a greater severity of COVID-19, such as: belonging to an indigenous population, a low socioeconomic level or living in the southern states of the country [14]. The lack of universal access to ancillary tests in the medical units, to quickly identify the probable cases of SARS-CoV-2, has led to the adoption of algorithms based on clinical data to help guide the decision-making process [7]. Correctly identifying patients with a high suspicion of infection by SARS-CoV-2 is paramount to the emergency services, for the control of infectious outbreaks [15, 16]. Some authors have developed predictive models for the diagnosis of the SARS-CoV-2 infection, to be used in settings where ancillary tests may not be available for first contact physicians [17–19]. However, the results of the studies published as of today are inconclusive, partly because of their great heterogeneity [10].

*Peyrony* et al. [20] carried out a prospective observational study in a French hospital involving 391 patients, out of whom 225 (57.66%) tested positive for SARS-CoV-2 by RT-PCR. In this group, 53 (23.6%) presented gastrointestinal symptoms such as vomiting, diarrhea, or abdominal pain; 147 (65.6%) had a temperature below 38 °C and 97 (43.3%) below 37.5 °C upon arrival at the emergency department. The symptomatic prevalence in the SARS-CoV-2 positive group was as follows: fever 176 (78.2%), cough 158 (70.2%), dyspnea 131 (58.2%), myalgia 71 (31.6%), rhinitis-pharyngitis 19 (8.4%), anosmia 31 (13.8%), headache 15 (6.7%), fatigue 34 (15.1%). Furthermore, the following symptoms were evaluated for their sensitivity and specificity: fever had a sensitivity of 78% and a specificity of 50%; dyspnea had a sensitivity of 32% and a specificity of 87%; anosmia had a sensitivity of 14% and a specificity of 98%; and oxygen saturation below 95% had a sensitivity of 17% and a specificity of 91% for the SARS-CoV-2 diagnosis.

*Tostmann* et al. [18], studied 803 health-worker patients through a questionnaire to evaluate their symptoms associated to COVID-19; 112 patients were positive for SARS-CoV-2 infection, out of which 90 answered the instrument. The analytical model that included all variables (general non-respiratory, respiratory, and gastrointestinal symptoms) excluding fever and cough, reached an AUC of 0.75 (95% CI:0.66–0.84), with a sensitivity of 82.4% and a specificity of 59.2%. The second analytical model that included symptoms significantly associated with the SARS-CoV-2 infection (3 or more symptoms) such as anosmia, myalgia, asthenia, headache, eye pain, and malaise, yielded a sensitivity of 91.2% and specificity of 55.6% for SARS-CoV-2 positivity.

*Salmon* et al. [21], performed a prospective, multicenter observational study at 5 hospitals in Paris to determine the frequency of SARS-CoV-2 positive patients with a loss of sense of smell; and to analyze the diagnostic accuracy of olfactory and gustatory dysfunction for the diagnosis of COVID-19. A total of 1824 patients were included in the second phase of the study, out of whom 849 (46.5%) tested positive for SARS-CoV-2. The positive predictive value (PPV) of olfactory and gustatory dysfunction was 78.5% (95% CI: 76.6–80.3%), with a sensitivity of 40.8% (95% CI: 38.5–43.0%), a specificity of 90.3% (95% CI: 88.9–91.6%), and a negative predictive value (NPV) of 63.6% (95% CI: 61.4–65.8%). Cough obtained a sensitivity of 70.4% (95% CI: 68.3–72.5%), a specificity of 32.4% (95% CI: 30.2–34.5%), a PPV of 47.5% (95% CI: 45.2–49.8%) and a NPV of 65.2% (95% CI: 53.5–58.0%).

In our series, we found a prevalence of SARS-CoV-2 infection of 53.72%. The most prevalent symptoms were: asthenia, headache and cough (63.36, 60.22% and 60. 08% respectively), similar to those reported in other series [18, 21, 22]. The symptomatology that was significantly associated to the SARS-CoV-2 infection was the presence of anosmia, with an OR of 3.23 ([95% CI: 2.52–4.17], *P* < 0.001), fever OR of 2. 98 ([95% CI: 2.47–3.58], *P* < 0.001), dyspnea OR of 2.9 ([95% CI: 2.39–3.51], *P* < 0.001), cough OR of 2.73 ([95% CI: 2.27–3.28], *P* < 0.001), SO2 < 93% OR of 2.73 ([95% CI: 1.89–4.00], *P* < 0.001) and myalgia with an OR of 1.66 ([95% CI:1. 39–1.98], *P* < 0.001); similar to what was reported by *Lan* et al. [22]. who found an OR of 6.5 (95% CI: 2.89–14.51) for anosmia, fever OR of 3.34 (95% CI: 2.07–5.41), myalgia OR of 2.41 (95% CI:1.50–3.89). Similarly, Tostmann et al. [18] reported an OR for anosmia of 23 (95% CI: 8.2–64.8), fever OR of 2.7 (95% CI: 1.7–4.2) and myalgia OR of 6.9 (95% CI: 4.2–11.3). In a multivariate model where symptomatology was adjusted according to other predictive variables, an OR for fever of 1.96 (95% CI: 1. 58–2.41], *P* < 0.001), cough OR of 1.95 ([95% CI: 1.58–2.41], *P* < 0.001), anosmia OR of 2.96 ([95% CI: 2.27–3.87], *P* < 0.001) and dyspnea OR of 1.48 ([95% CI: 1.17–1.87], *P* < 0.001) were found.

Combining cough-fever and cough-anosmia resulted in an OR of 2.79 ([95% CI: 2.12–3.69], *P* < 0.001) and 2.48 ([95% CI: 1.65–3.74, *P* < 0.001) respectively; something similar to what was reported by *Lan* et al. [22].

In our study, a symptomatology model was created where 6 symptoms were combined obtaining for ≥2 symptoms, a sensitivity of 83.45% (95% CI: 81.17–85.55%) and a specificity of 32.86% (95% CI: 29.94–35.89%); and an association with the presence of SARS-CoV-2 with an OR of 2.46 ([95% CI: 2.00–3.04, *P* < 0.001). Similarly, *Tostmann* et al. reported a sensitivity of 91.2% and a specificity of 55.6%, considering a cut-off point for 3 or more symptoms [18].

The findings of our study suggest that the symptomatology (anosmia, fever, dyspnea and cough) by itself have a close relationship with the presence of the SARS-CoV-2 infection. Given the symptomatology complexity presented in this type of condition, the combination of symptoms, reported in different series, allows for a greater accuracy in the presumptive diagnosis of the SARS-CoV-2 infection (fever, cough, dyspnea, anosmia, SO2 < 93%). Finally, the combination of symptoms significantly associated to SARS-CoV-2 infection, integrated in a predictive model, will allow for a faster and more accurate final diagnosis, when limited ancillary resources are available.

From the physio-pathological viewpoint, the presence of dyspnea and hypoxemia has its explanation in the lung damage caused by the virus. The high expression of ACE2 in the apical lung cells [23], promotes adhesion, penetration and destruction of lung tissue, causing a diffuse interstitial and alveolar inflammatory exudate production, as well as edema [24]. Regarding anosmia, some theories have emerged based on the findings of the neuro-invasion mechanism of SARS-CoV and MERS-CoV, due to the great genetic similarity that these viruses have with SARS-CoV-2 (89.1% similarity with SARS-CoV) [25, 26]. Stemming from the above-mentioned, three routes of SARS-CoV-2 invading the nervous system have been proposed: 1) the hematogenous route, 2) the direct route (through the cribriform plate via the olfactory neuro epithelium) and 3) a retrograde axonal transport to the central nervous system [1, 27, 28]. On the other hand, it has been hypothesized that the increase of bradykinins (secondary to SARS-CoV2 infection), specifically DABK, activates the BK1 receptors of the centers in charge of the sense of taste and smell located in the medulla oblongata, which results in the alteration of these senses [29, 30].

The strengths of this study include its contribution to the world’s information around the prevalence of the symptomatology in patients with COVID-19, studied in a significant amount of symptomatic patients, which allows for the findings and inferences to be relevant; its prospective nature improving its internal validity.

Some of our limitations were: the use of RT-PCR as a reference test, since its diagnostic performance has not been accurately determined [31] and its inherent technical limitations [32]. Some authors have reported a low sensitivity, such as *Wang* et al. who obtained a sensitivity of 60% for the detection of SARS-CoV-2 in nasopharyngeal exudate samples [5]. Another limitation was not including gastrointestinal symptoms and taste alterations, which have been reported in other studies [20, 33]. More prospective studies regarding the symptomatology associated to COVID-19, that weigh in the symptomatology strategy in the diagnosis of the disease, are required; and finally, the non-inclusion of the 880 patients who did not undergo the SARS-CoV-2 RT-PCR.

## Conclusion

The combination of ≥2 symptoms / signs (fever, cough, anosmia, dyspnea and oxygen saturation < 93%, and headache) results in a highly sensitivity model for a quick and accurate diagnosis of COVID-19, and should be used in the absence of ancillary diagnostic studies. Symptomatology, alone and in combination, may be an appropriate strategy to use in the emergency department to guide the behaviors to respond to the disease.

## Data Availability

The datasets used and/or analyzed during the current study are available from the corresponding author on reasonable request. Authors have gained informed consent for publication of the dataset from participants at the point of recruitment to the trial.

## Abbreviations

WHO: World Health Organization
COVID-19: Coronavirus disease 19
SARS-CoV-2: severe acute respiratory syndrome coronavirus 2
LR +: positive likelihood ratio
LR-: negative likelihood ratio
AUC: area under the curve
IQR: interquartile range
SD: standard deviation
OR: odds ratio
95% CI: 95% confidence interval
SO2 < 93%: saturation of less than 93%
PPV: positive predictive value
NPV: negative predictive value
